# Relationship among Photopic Negative Response, Retinal Nerve Fiber Layer Thickness, and Visual Field between Normal and POAG Eyes

**DOI:** 10.1155/2013/182021

**Published:** 2013-02-17

**Authors:** Xiaoli Shen, Lina Huang, Ning Fan, Jing He

**Affiliations:** Shenzhen Eye Hospital, Medical College of Jinan University, 18 Zetian Road, Futian District, Shenzhen 518000, China

## Abstract

*Purpose*. To determine the relationship among photopic negative response (PhNR) of the electroretinogram (ERG), retinal nerve fiber layer (RNFL) thickness, and the visual field in normal and glaucomatous patients. *Methods*. Thirty-eight normal volunteers and one hundred twenty-four patients with Primary open-angle glaucoma (POAG) were enrolled in the study. The PhNRs were elicited by white stimuli on a white background and red stimuli on a blue background. The visual field parameters were measured using the standard automated perimetry (SAP). The spectral domain optical coherence tomography (SD-OCT) was used to measure the retinal nerve fiber layer (RNFL) thickness around the optic disc. *Results*. The PhNR amplitude (W/W, B/R), MD, and mean RNFL thickness in POAG eyes were significantly lower than normal eyes (*P* = 0.001). The *R* value in Normal + Glaucomatous group was higher than that of the only glaucomatous group. The *R* values of PhNR amplitude (B/R) with MD and RNFL were higher than those of PhNR amplitude (W/W). Significant linear association was found in the relationship between RNFL thickness and PhNR amplitude (B/R) (*R* ^2^ = 0.5, *P* = 0.001). However, significant curve associations were found in the relationship between MD and PhNR amplitude (B/R) and RNFL thickness (*R* ^2^ = 0.525, 0.442, *P* = 0.001). *Conclusions*. The ganglion cell activity can be more efficiently evaluated with the PhNR elicited with a red than with a broadband stimulus. The linear relationship between the PhNR amplitude and RNFL thickness indicates that inner retinal function declines proportionately with neural loss in glaucomatous eyes. The PhNR and RNFLT are more objective tools to detect glaucomatous damage than visual field.

## 1. Introduction 

 Glaucoma is an optic neuropathy characterized by progressive loss of retinal ganglion cells (RGCs) and their axons, changes in optic disc topography, and associated deficits of visual function. Early detection is important to initiate treatment in the earliest phases of glaucoma and to avoid its natural progression to blindness.

 Various techniques for functional and structural evaluation of the neuroretinal structures of the ocular system are available. It was generally believed that the neural activity of retinal ganglion cells (RGCs) contributes little to shaping the ERGs. However, a response driven by RGCs receiving signals from cones was newly discovered and called the PhNR [1]. The PhNR is strongly attenuated in primates with experimentally induced glaucoma [1] and after an intravitreous injection of tetrodotoxin that blocks the voltage-gated sodium channels in retinal neurons including RGCs, amacrine cells, cone bipolar cells, and cones [2–4].

In the clinic, the PhNR amplitude is reduced in patients with primary open-angle glaucoma (POAG), and the decrease in amplitude correlated with the degree of optic nerve damage reprinted by retinal nerve fiber layer (RNFL) thickness and visual field loss [5]. A red stimulus light on a blue backgroud was used to elicitnd the PhNR in the previous studies, whereas a white stimulus on a white background is recommended by the International Society for Clinical Electrophysiology of Vision to produce cone ERG [6].

Cirrus OCT provide objective and quantitative measurements that is highly reproducible and show good agreement with optic nerve head structure and visual function [7]. The retinal nerve fiber layer thickness (RNFLT) measured by optical coherence tomography (OCT) correlated highly with the amplitudes of the PhNR in eyes with optic nerve atrophy [8]. This association indicates that the amplitude of the PhNR is a good measure of the decrease in the RNFL thickness in patients with optic nerve atrophy.

 It was well known that anatomic changes in RGCs can precede the functional loss of RGCs. Earlier studies [9] have shown that up to 30–50% of nerve fibers may be lost before a visual field loss can be observed, meaning that anatomical changes precede the functional damage.

The purpose of this study was to evaluate the clinical significance of the PhNRs recorded from eyes with POAG by correlating the amplitudes of the PhNR with the RNFLT, and mean deviation (MD) is obtained in glaucomatous eyes by the newer instruments.

## 2. Methods

### 2.1. The Subjects

 One hundred and twenty-four eyes of 124 POAG patients (including of early, advanced, and end-stage patients) were recruited. Their ages ranged from 19 to 75 years with a mean ± SD of 45.2 ± 15.9 years. POAG was defined when the intraocular pressure was >21 mmHg together with characteristic glaucomatous optic disc changes and corresponding visual field defects measured by static perimetry and an open angle by gonioscopy. Secondary glaucoma (e.g., uveitic glaucoma) was excluded. Visual field defects were identified [10] when (1) the pattern deviation plot showed a cluster of ≥3 nonedge points that had sensitivities less than that of the lower 95% centile ranges (*P* < 0.05) with at least one less than the lower 99% centile range (*P* < 0.01), (2) the value of the corrected PSD was less than the lower 95% centile range (*P* < 0.05), or (3) the Glaucoma Hemifield Test was outside the normal limits. 

Thirty-eight eyes of 38 age-matched normal volunteers (range 20–73 years, mean ± SD of 43.1 ± 14.5 years) were included. All subjects underwent full ophthalmic examination, including visual acuity, refraction, intraocular pressure measurement with Goldmann tonometry, dilated fundus examination with stereoscopic biomicroscopy of optic nerve head under slit-lamp, and indirect ophthalmoscopy. Inclusion criteria were best-corrected visual acuity of not worse than 20/40 and spherical refractive error within the range of −6.00 DS to +3.00 DS. Subjects were excluded if they had history of retinal disease, surgery, laser procedures, diabetes mellitus, or neurologic disease.

### 2.2. Photopic ERG Recordings

 The photopic ERGs were elicited by white stimuli and red stimuli of 2 cd*·*s/m^2^ on white background and blue background of 25 cd/m^2^. The recording condition is the standard for clinical ERG [7]. The stimulus and background lights were produced by light-emitting diodes (LEDs). ERGs were recorded simultaneously from both eyes. Before the recordings, all subjects were light adapted to the background light for at least 10 minutes. The Pupils were maximally dilated to 8.0 mm by 1% tropicamide. After light adaptation, a corneal electrode was applied under topical anesthesia with 0.5% proparacaine (Alcaine, Alcon-Couvreur, Puurs, Belgium). One percent methyl cellulose was spread over the surface of the electrode to prevent mechanical damage to the cornea. The reference electrode was placed on the ipsilateral temple, and a ground electrode was positioned to the center of the forehead. The duration of the stimulation was limited to less than five milliseconds (ms) and the intensity and duration were controlled by an electronic stimulator. The interval between the stimuli was greater than two seconds. Electrical signals from the electrode were amplified 1000 times and were digitalized using an analog-to-digital converter. High and low cutoff limits for the signals were set at 300 Hz and 1.0 Hz, respectively. The averaged results from five repeated measurements were analyzed in this study. 

**Figure 1:**
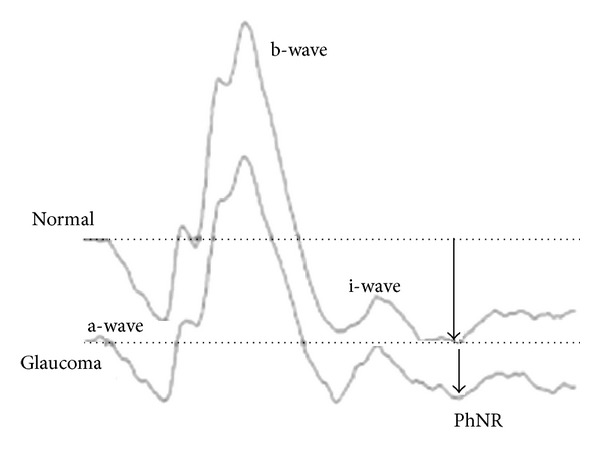
Representative ERGs recorded from a normal and a glaucomatous eye with moderate defects in the visual fields.

The PhNR amplitude was defined as the difference between the baseline and the peak of the negative wave following the i-wave (Figure 1). The PhNR amplitude (W/W, B/R) was compared between the normal and glaucoma group.

### 2.3. Visual Field Analyses

The Humphrey Visual Field Analyzer (Model 750-II, Humphrey Instruments, Carl Zeiss Meditec, Dublin, CA, USA) SITA standard 24-2 strategy was used to evaluate the visual field. The measurement of the visual field was made after at least 5 minutes of adaptation to the background lights. The MD was defined as the mean of the differences between the measured sensitivity and normal values of age-matched control eyes. Thus, the MDs represented the depression of sensitivity over the whole visual field.

 When the fixation losses were >20%, the false-positive and false-negative rates >15%, the visual field was considered to be unreliable and excluded from the analysis. The interval between the visual field testing and ERG recording was less than 1 month.

### 2.4. Spectral Domain Optical Coherence Tomography (SD-OCT)

The RNFL thickness around the optic nerve head was imaged by the Cirrus SD-OCT (software version 5.0, Carl Zeiss Meditec, Dublin, CA, USA) using the optic disc cube scan. It is composed of 200 × 200 A scans covering an area of 6 × 6 mm^2^ centered on the optic nerve head. The total scan time was 1.48 seconds. The requirement for image quality was signal strength >6. The parameter, average RNFL thickness, was obtained and analyzed.

### 2.5. Statistical Analysis

 The significance of the differences in age, refraction, gender, MD, Avg-RNFLT, and PhNR amplitude was determined by independent-sample *t*-test, Pearson's coefficient functional, and morphologic parameters. The Deming regression was also used to evaluate the association between these parameters. In all statistical analyses, *P* < 0.05 was considered statistically significant. All analyses were performed using commercial software SPSS 17.0.

## 3. Results

### 3.1. The Data Comparison between Normal Group and POAG Group

The mean age, refractive error, and gender distribution were not significantly different between the normal and POAG groups (*P* > 0.05) (Table 1).

 In the normal group, the MD, Avg-RNFL thickness, and PhNR amplitude (W/W, B/R) were −1.23 ± 1.17 dB, 101.7 ± 10.9 *μ*m, and 45.6 ± 10.9 uV, 47.8 ± 10.7 uV, respectively. In the POAG group, the MD, Avg-RNFL thickness, and PhNR amplitude (W/W, B/R) were −10.66 ± 9.85 dB, 69.9 ± 14.0 *μ*m, 26.5 ± 12.7 uV, and 27.2 ± 13.5 uV, respectively.

 The MD, Avg-RNFL thickness, PhNR amplitude (W/W, B/R) were significantly reduced in the POAG group compared to that of the normal group (*P* = 0.001).

### 3.2. The Correlation between the PhNR and Other Parameters

The R value in Normal + Glaucoma group and Glaucoma group was higher than that of the only glaucomatous group. The *R* value with PhNR (B/R) was higher than PhNR (W/W) (*P* < 0.05) (Table 2). The trend of the relationship was similar in the two groups.

**Figure 2:**
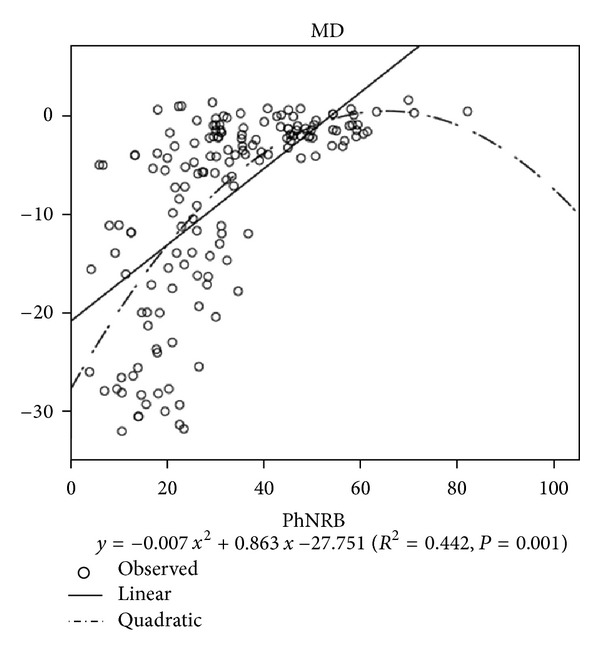
PhNR amplitudes (B/R) against MD.

In Normal + Glaucoma group and Glaucoma group, the reduction of the PhNR amplitude correlated significantly with the decrease in the MD (Figure 2). The correlation coefficients were higher when the PhNR amplitude (W/W, B/R) was expressed in quadratic units than linear units (Table 2, Quadratic *R* = 0.682, 0.660, 0.604, and 0.564; Linea *R* = 0.632, 0.580, 0.570, and 0.498; *P* < 0.05).

**Figure 3:**
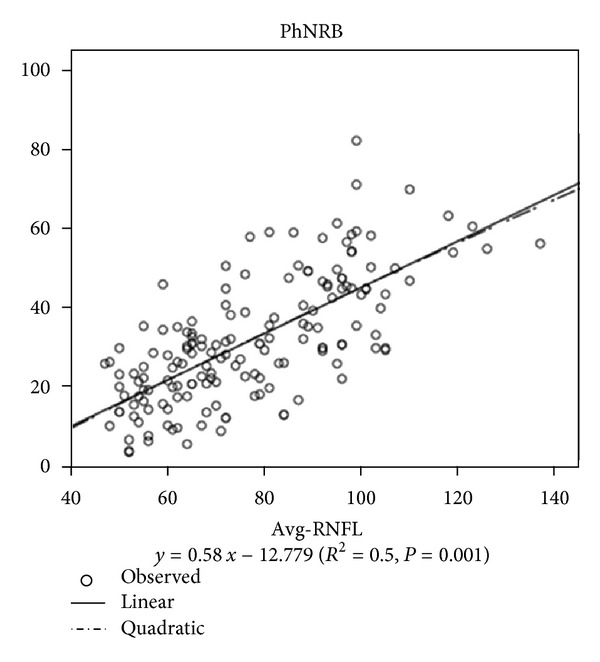
PhNR amplitudes (B/R) against Avg-RNFLT.

 In Normal + Glaucoma group and Glaucoma group, the correlation between the PhNR amplitude and RNFLT measured by SD-OCT was shown in Figure 3. The PhNR amplitude (W/W, B/R) correlated linearly with the Avg-RNFLT (Table 2, Linear *R* = 0.627, 0.707, 0.454, and 0.565).

**Figure 4:**
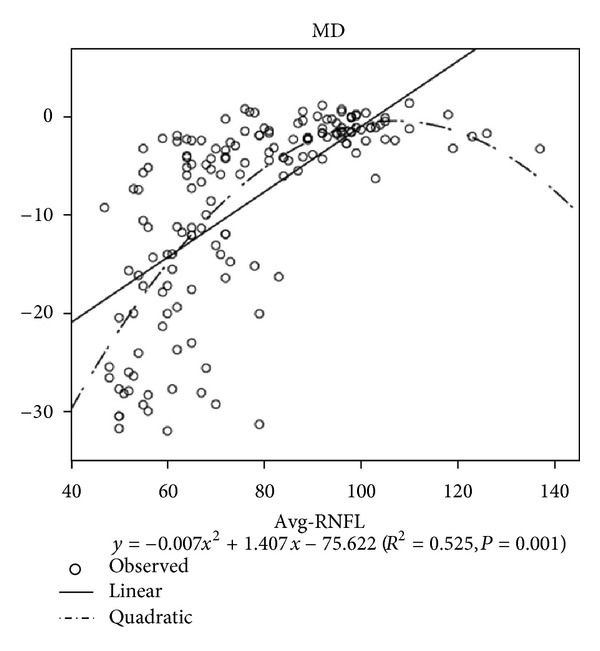
Avg-RNFLT against MD.

The correlation between the Avg-RNFLT and MD was shown in Figure 4. The reduction of the Avg-RNFLT correlated significantly with the decrease in the MD. The correlation coefficients were higher in quadratic units than in linear units (Table 2, Quadratic *R* = 0.725, 0.654; Linear *R* = 0.666, 0.630; *P* < 0.05).

## 4. Discussion

 Glaucoma is characterized by progressive degeneration of the optic nerve and loss of retinal ganglion cells (RGCs) [11]. Viswanathan et al. [1] compared the ERG findings between normal eyes and tetrodotoxin-injected eyes in monkeys. The results of their study showed that the PhNR amplitude was reduced in the tetrodotoxin-injected eyes. In addition to this experimental evidence, it has been demonstrated that the PhNR was reduced in patients with optic nerve and retinal diseases that affect mainly the RGCs and retinal nerve fiber layer [7, 8]. In our study, the PhNR amplitude was reduced in the POAG group compared to the normal group. These findings are similar to those reported by other studies [12, 13]. PhNR amplitude in glaucomatous eyes was outside the normal range of normal control eyes, which suggested PhNR amplitude measurement can be used to differentiate diseased eyes from normal eyes. In addition, the reduction of the PhNR amplitude not only correlated with the degree of visual field defect but also with the neural loss assessed by anatomic structure of RNFL [14–16].

 Rangaswamy et al. [17] were the first to show that monochromatic stimuli (a red flash on a blue background) can elicit larger PhNR than the broadband stimuli (a white stimulus on a white background). They reasoned that these combinations of monochromatic stimuli were more specific for a single cone type, and therefore they induced less spectral antagonism in the receptive fields of distinct ganglion cell populations. They also have found that the glaucomatous ganglion cell dysfunction affects the PhNR to red stimulus on a blue background similarly the PhNR to white stimulus on a white background. In order to make a better comparision, we set a same stimuli and background parameters. In our study, we show a greater PhNR reduction with monochromatic red than with white stimulus in glaucoma patients. In addition, the PhNR to red stimulus on a blue background correlated better with RNFL and MD than the PhNR to white stimulus on a white background did; these findings suggested that ganglion cell activity can be more efficiently evaluated with the PhNR elicited with a red than with a broadband stimulus.

### 4.1. Correlation of PhNR with MD of Visual Field

Viswanathan et al. [5] were the first to show that the PhNR amplitude correlated linearly with the MD determined by the static visual field tests. We attempted to confirm their results in a larger number of patients and a wider range of disease stages. However, we found some differences from their results. In plotting the PhNR amplitude against the MD, we found that a curvilinear regression was a better fit than a linear regression. When the PhNR amplitude was plotted on a quadratic scale, the correlation coefficients were higher than with a linear scale. This is not the first study to show a curvilinear relationship between the PhNR amplitude and visual sensitivity obtained by visual field testing. It has been demonstrated that the PhNR amplitude correlated with MD in a curvilinear fashion. The correlation coefficients were higher when the PhNR amplitude was expressed in logarithmic units rather than linear units [14].

The curvilinear association of the PhNR with the MD (dB) indicated that large changes of the PhNR amplitude correspond with small loss of the MD, and maybe the MD could still be in the normal range. 

### 4.2. Correlation of the PhNR with RNFL of Structural Parameter

In our study, the PhNR amplitude correlated linearly with RNFL thickness of structural parameter, indicating that the function of RGCs declines proportionately with the structure of RGCs loss in glaucoma. These findings suggested that compensational mechanisms do not work at the RGC level in glaucomatous eyes. Ours is not the first study to find a linear relationship between electrical signals from RGCs and anatomic structure of the RNFL. Machida et al. [14] demonstrated that the PhNR amplitude correlated linearly with RNFLT (*R* = 0.58); the results is similar to our study. In addition, Toffoli et al. [15] found that a significant linear correlation was observed between the RNFL thickness and the pattern ERG amplitude in glaucomatous patients. In the past study, the PhNR amplitude correlated highly with the RNFLT measured by OCT in patients with optic nerve atrophy induced by trauma, compression, and optic neuritis [8]. These findings indicated that PhNR is a good measure of the surviving ganglion cells and their axons of optic nerve disease.

We measured the averaged RNFLT around the optic nerve head because the PhNR is supposed to reflect the function of RGCs throughout the ocular fundus. Measuring the PhNR and RNFL seems more suitable for the optic nerve diseases in which RGCs are diffusely affected than for glaucoma at early and moderate stages when RGCs are locally impaired.

### 4.3. Correlation of the RNFL with MD

The loss of retinal ganglion cells in glaucoma can be reflected structurally as localized or diffused thinning of the retinal nerve fiber layer, and its measurement has been related with the functional damage in visual field (VF) [18, 19]. SD-OCT (Carl Zeiss Meditec, Dublin, CA, USA) was the latest commercially available imaging modality designed to measure RNFL thickness. A number of recent studies have reported high correlations between VF sensitivity and RNFL thickness in glaucoma. Leung et al. [20] showed that the coefficient of correlation between VF mean deviation (MD) and average RNFL thickness was 0.79. Although statistically significant correlations were found in these studies, the structure/function relationship was primarily investigated with linear regression analysis, which may not be an adequate model to describe and fit the nonlinear portion of the relationship. Comparisons with linear and nonlinear regression models are essential to identify and confirm the precise nature of the structure/function relationship. The regression function would then be useful for understanding the trend and pattern of disease progression and for selecting an appropriate monitoring strategy to detect the changes. In fact [21], when VF sensitivity MD (dB) was plotted against RNFL thickness, the third- and second-order polynomial models fit significantly better than the linear model in the GDx VCC and the Stratus OCT RNFL measurements, respectively. The structure/function relationship was better explained with nonlinear models when visual sensitivity in MD (dB) was plotted against RNFL thickness (Figure 4).

 The visual field is a subjective test, depending on the patient's training and collaboration. On the contrary, the PhNR and the SD-OCT are objective, patient-independent tests. This study represents clinical evidence *in vivo* of the relevant relationship that exists between the anatomical and the functional objective aspects of optic nerve. The PhNR seems to represent a useful additional tool to detect glaucomatous damage, but further studies are needed to extend the clinical use of the technique.

## 5. Conclusion

 The PhNR amplitude was linearly correlated with structural parameters of the RNFL, and thus the PhNR could be a measure for tracking the morphologic state of the optic disc and RGC axons. Linear structure-function correlations suggested that inner retinal function declines proportionately with neural loss in glaucoma.

## Figures and Tables

**Table 1:** Demographic and clinical data for this study.

	Normal	Glaucoma	*P* value
Number of subjects	38	124	
Gender (male/female)	25/13	73/51	0.566*
Age (yrs) mean ± SD	43.1 ± 14.5	45.2 ± 15.9	0.152
Refraction (D) mean ± SD	−0.97 ± 1.97	−1.25 ± 1.97	0.525
Visual field MD (dB) ± SD	−1.23 ± 1.17	−10.66 ± 9.85	0.001
Average RNFL thickness (Cirrus OCT) (*μ*m) mean ± SD	101.7 ± 10.9	69.9 ± 14.0	0.001
PhNR amplitude (W/W)	45.6 ± 10.9	26.5 ± 12.7	0.001
PhNR amplitude (B/R)	47.8 ± 10.7	27.2 ± 13.5	0.001

D: diopters; MD: mean deviation; SD: standard deviation; PhNR: photopic negative response; W/W: white stimulus and white background; B/R: red stimuls and blue background; P*: Chi-square test*; P*: independent-sample* t*-test.

**Table 2:** The relationship among the PhNR, MD and Avg-RNFL in normal and glaucoma groups.

	Normal + Glaucoma (*n* = 162)		Glaucoma (*n* = 124)	
	*R* (Linear)	*R* (Quadratic)	*R* (Linear)	*R* (Quadratic)
RNFL versus PhNR (W/W)	0.627	0.628	0.454	0.454
RNFL versus PhNR (B/R)	0.707	0.707	0.565	0.568
RNFL versus MD	0.666	0.725	0.630	0.654
MD versus PhNR (W/W)	0.580	0.660	0.498	0.564
MD versus PhNR (B/R)	0.632	0.682	0.570	0.604

All the *P* < 0.05.
